# A Novel TCN-LSTM Hybrid Model for sEMG-Based Continuous Estimation of Wrist Joint Angles

**DOI:** 10.3390/s24175631

**Published:** 2024-08-30

**Authors:** Jiale Du, Zunyi Liu, Wenyuan Dong, Weifeng Zhang, Zhonghua Miao

**Affiliations:** 1 College of Electromechanical Engineering, Qingdao University of Science and Technology, Qingdao 266000, China; 2022030016@mails.qust.edu.cn (J.D.); 4022030040@mails.qust.edu.cn (Z.L.); dwyxn1314@163.com (W.D.); 2 School of Mechatronic Engineering and Automation, Shanghai University, Shanghai 200072, China; zhhmiao@shu.edu.cn

**Keywords:** surface electromyography (sEMG), wrist kinematics estimation, human–machine interaction (HMI), temporal convolution network (TCN), long short-term memory neural network (LSTM)

## Abstract

Surface electromyography (sEMG) offers a novel method in human–machine interactions (HMIs) since it is a distinct physiological electrical signal that conceals human movement intention and muscle information. Unfortunately, the nonlinear and non-smooth features of sEMG signals often make joint angle estimation difficult. This paper proposes a joint angle prediction model for the continuous estimation of wrist motion angle changes based on sEMG signals. The proposed model combines a temporal convolutional network (TCN) with a long short-term memory (LSTM) network, where the TCN can sense local information and mine the deeper information of the sEMG signals, while LSTM, with its excellent temporal memory capability, can make up for the lack of the ability of the TCN to capture the long-term dependence of the sEMG signals, resulting in a better prediction. We validated the proposed method in the publicly available Ninapro DB1 dataset by selecting the first eight subjects and picking three types of wrist-dependent movements: wrist flexion (WF), wrist ulnar deviation (WUD), and wrist extension and closed hand (WECH). Finally, the proposed TCN-LSTM model was compared with the TCN and LSTM models. The proposed TCN-LSTM outperformed the TCN and LSTM models in terms of the root mean square error (*RMSE*) and average coefficient of determination (*R*^2^). The TCN-LSTM model achieved an average *RMSE* of 0.064, representing a 41% reduction compared to the TCN model and a 52% reduction compared to the LSTM model. The TCN-LSTM also achieved an average *R*^2^ of 0.93, indicating an 11% improvement over the TCN model and an 18% improvement over the LSTM model.

## 1. Introduction

The wrist, as a complex joint connecting the hand and forearm in the upper limb of the human body, undertakes the responsibility of performing various fine motor actions in daily life, a fact that is well recognized by many. However, worldwide, about fifteen hundred people suffer from strokes each year, and nearly half of them will have permanent impairment of their ability to exercise and require therapeutic services [1,2]. The progress of upper-limb rehabilitation robots has provided this group of people with improved mobility assistance [3,4]. In particular, in the early phase of patient rehabilitation, the upper-limb rehabilitation robot can mechanically enable the patient’s affected limb to perform a certain range of activities, which is also known as the passive mode. Once the patient has a certain level of self-motor capacity, an initiative rehabilitation method is required, with the fundamental difficulty being how to accurately extract and interpret human–robot synergy information [5]. One of the important bioelectrical signals is the surface electromyographic (sEMG) signal, which is the result of a combined temporal and spatial superposition of sequences of action potentials emitted by multiple motor units presented on the surface of the skin [6]. In particular, sEMG signals can be detected approximately 50–100 ms before limb motion. Utilizing valuable information from action-preceding electromyographic signals, it is possible to convert them into control signals for mechanical hands or prosthetics, thereby facilitating proactive therapeutic interventions for patients [7,8,9]. In addition, sEMG signals have the advantages of easy measurement, high temporal validity, and high fidelity, and have gradually become a widely used and highly efficient biosignal in the field of upper-limb rehabilitation robotics [10].

In the past few years, researchers have designed a variety of methods to extract human motion intention from sEMG signals, which can be classified into two main categories [11]. One is to label individual discrete movements of human beings and obtain category outputs through classifiers, which can be directly used as driving signals for manipulators or prosthetics. The other is to decode the human motion information into continuous estimates (e.g., the force at each moment, the angle of joint motion, etc.). The former was put into research earlier than the latter. Cai et al. [12] proposed the application of support vector machine (SVM) classifiers to the rehabilitation robot ReRobot and the use of time-dependent multi-feature sets to implement a mirror rehabilitation strategy where the robot can actuate the movement of the affected side by recognizing the movement of the patient’s healthy side. Park et al. [13] introduced a user-adaptive model leveraging a convolutional neural network (CNN) for sEMG-based gesture recognition, marking the pioneering application of deep learning in this domain. The model demonstrated superior performance in classification accuracy and robustness compared to the conventional SVM model. Although discrete motion estimation research has matured, these classifier-based methods can only identify a single simple intention and are suitable for cerebral palsy patients in the early stages of recovery, while continuous motion estimation is more flexible and compensates for this limitation [14,15].

Traditionally, many continuous motion estimation methods have utilized conventional machine learning algorithms to interpret sEMG signals and conduct feature selection. However, with the advancement of deep learning, more advanced networks are being incorporated into prediction models to better extract features from historical time series data and improve the accuracy of traditional models. In the study of continuous motion estimation based on sEMG signals, the CNN model has become one of the most popular architectures because of its ability to parse features from multi-channel sEMG signals layer by layer. Ameri et al. [16] used the CNN model for myoelectric control and found that the advantage of the regression prediction task, which is different from the motion classification task, is that motion can be controlled independently and in real time. Hajian et al. [17] proposed a TS-CNN model that uses different scales to extract features from the original sEMG signals to estimate elbow joint motion angles and obtained an average *R*^2^ value of 0.77. The CNN model could identify local feature correlations [18], but it could not effectively capture long-term dependent information for time-dependent signals such as sEMG signals. Therefore, based on CNN, Bai et al. [19] proposed a temporal convolutional network (TCN) to make up for this defect. TCN networks utilize causal dilated convolution to achieve a significantly expanded receptive field and minimize information loss. Additionally, the integration of residual connections helps circumvent issues such as gradient disappearance or explosion. Researchers have effectively applied TCNs to sEMG domains. For example, Liu et al. [20] introduced the SE-TCN model for estimating upper-limb-motion angles, which enhances the depth and breadth of the TCN network. Chen et al. [21] conducted a study on the impact of the depth of the TCN network and the size of convolution kernel on prediction accuracy. They proposed a large-scale convolution approach to estimate the angles of multiple joints in grasping motions and achieved an impressive accuracy of 71.6% on the NINA dataset. The LSTM model, based on the architecture of recurrent neural networks (RNNs), also demonstrated good performance in time series estimation tasks. Tang et al. [22] optimized the LSTM model using particle swarm optimization (PSO) and achieved an *RMSE* of 0.1025 in the continuous wrist estimation experiment. To mitigate noise interference, Zhang et al. [23] proposed the closed-loop L-NTZNN model, which combines the LSTM network and sEMG, and verified its performance using the Kruskal–Wallis method (*p* > 0.05). Additionally, Ma et al. [24] introduced the SCA-LSTM algorithm, integrating a short-link autoencoder with LSTM to reduce noise interference. However, the linear processing method resulted in a significant sacrifice in training time. Lin et al. [25] proposed a RoFormer-based cross-subject model to continuously estimate finger kinematics and extended it to new subjects using an adversarial transfer learning (ATL) approach. However, this study purposefully eliminated the inaccurate data from Ninapro, which diminished the validity of the results. Wang et al. [26] proposed a continuous angle decoding model building scheme that estimates hand angles and demonstrated that the ensemble learning model based on the tree model performed significantly better in processing tabular data than traditional machine learning methods such as the Gaussian process.

Inspired by the observations mentioned above, a hybrid TCN-LSTM framework was proposed to combine the advantages of the two networks and take full advantage of the spatiotemporal correlation of sEMG signals. Specifically, the TCN extracts deep features from multi-channel sEMG signals, and then LSTM captures long-term dependencies to accurately construct the mapping relationship between the sEMG signals and wrist angles. Compared with the single model, the hybrid CNN-LSTM model had stronger robustness. We demonstrated the proposed model’s ability to predict the wrist angle using the Ninapro dataset. We plotted predicted angle graphs alongside corresponding actual angle graphs and evaluated the performance of the proposed model and comparative models using *RMSE* and *R*^2^.

The remainder of this article is organized as follows: Section 2 introduces the dataset used and describes the structure and principles of the proposed TCN-LSTM hybrid model. Section 3 presents the estimation results and performance comparisons between the proposed model and two other contrastive models, followed by detailed discussions. In Section 4, comprehensive conclusions and future research trends are provided.

## 2. Materials and Methods

### 2.1. Dataset

We used a publicly available dataset called Non-Invasive Adaptive Prosthetics (Ninapro) to validate the proposed scheme. To date, a total of ten datasets (DB1~DB10) have been curated, comprising physiological metrics from 180 trans-radial amputees and intact subjects (i.e., electromyographic signals, kinematics, inertial measurements, etc.).

**Figure 1:**
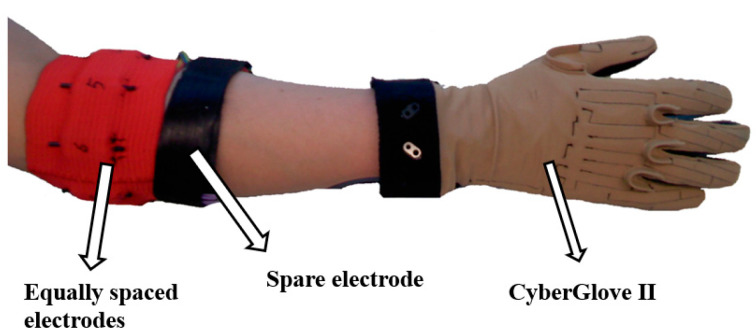
The equipment for recording experimental data.

As shown in Figure 1, the devices used in this dataset to record sEMG signals and motion data of the hand were the OttoBock myograph and the CyberGlove II data glove, respectively. The OttoBock consists of 10 MyoBock 13 E200-50 electrodes, of which 8 electrodes were placed uniformly underneath the elbow joint, and the remaining 2 electrodes were placed on the flexor and the extensor muscles. The raw sEMG signals were also amplified, filtered, and rectified. The CyberGlove II uses specialized resistive bending sensing technology to accurately detect 22 angle data points in real time using 22 sensors. The sEMG and angle data were both sampled at a frequency of 100 Hz [27,28].

**Figure 2:**
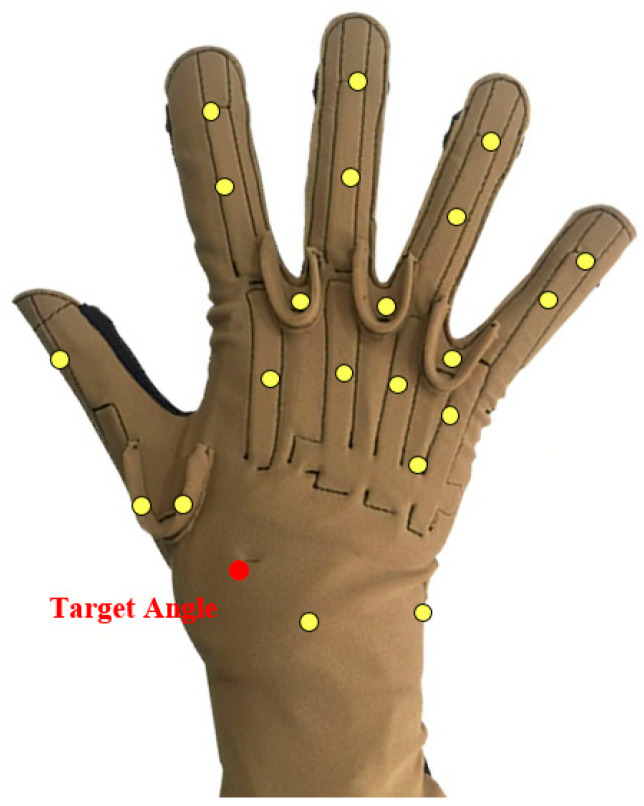
The target angle we selected for experiments.

**Figure 3:**
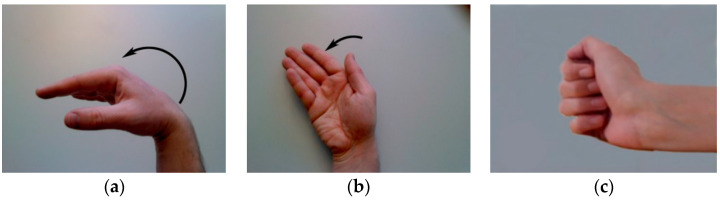
The three types of movement we selected: (**a**) wrist flexion (WF); (**b**) wrist ulnar deviation (WUD); (**c**) wrist extension and closed hand (WECH).

Reference [21] used data from eight participants in DB2 at random to estimate the continuous motion of the grasping action, and Reference [29] only used data from the first five subjects in DB5 to complete the finger joint angle estimation. This is because selecting a small number of subjects that include as much information about all the subjects as possible, rather than using all subjects’ data directly, reduces the computational cost of model training. Therefore, we selected the first eight subjects from Ninapro DB1, and Table 1 provides some detailed information about these subjects. The wrist, as a complex joint in the upper limb of the human body that connects the hand and forearm, is responsible for performing various fine motor activities in daily life. Therefore, we selected three types of movements closely related to the wrist from DB1, as shown in Figure 2. These movements included wrist flexion (WF), wrist ulnar deviation (WUD), and wrist extension closed hand (WECH). Figure 3 illustrates the target angles selected to effectively capture angle data for these three movement types.

### 2.2. Pre-Processing

The sampling frequency of the sEMG signal and kinematic data of the Ninapro DB1 was 100 Hz. In addition, because the original signal has been filtered and rectified, we did not need to repeat the operation of the data set to directly apply it. Considering the strong nonlinearity and short-time validity of sEMG, it is necessary to segment the sEMG data and extract the feature information of each part. Specifically, we divided the data for all subjects in DB1 into windows of with a length of 100 (0.1 s) with 10 (0.01 s) overlaps, and the next data point *k +* 1 of the last data point *k* of the recording window was recorded as the observed value for that window.

The time domain method, frequency domain method, and time–frequency domain method are the most commonly utilized techniques for sEMG analysis. Reference [29] chose six features that can be classified into temporal and frequency domains. The time domain group consisted of Mean Absolute Value (MAV), Root Mean Square (RMS), and Variance (VAR). In comparison, the frequency domain group consisted of Mean Power (MNP), Mean Frequency (MNF), and median frequency (MDF). Every feature in each domain was used independently. The ideal features for their study were then identified by comparing the performance of each feature. We tested the six features mentioned in their work using our experimental settings, and the results are shown in Table 2. The frequency domain features performed slightly better, but required threefold more training time. The *RMSE* and *R^2^* scores of MAV and RMS were in the middle-upper level among the six features, while their training times were the shortest. In terms of continuity and real time capability, the time-domain features performed better than the frequency domain and time-frequency domain features [30]. We ended up using two time-domain features representing muscle fatigue and activity levels, MAV and RMS, as the feature for extraction. This reduces the time spent on feature extraction while ensuring that complete information is extracted. These are the two best features that best represent the specific characteristics that the author used [21,31]. The equations are as follows:

(1) $$MAV=\frac{1}{N}\sum_{i=1}^{N}\left|x(i)\right|$$

and

(2) $$RMS=\frac{1}{N}\sum_{i=1}^{N}x^{2}(i)$$

### 2.3. Evaluation Metric

To evaluate the prediction performance of the proposed method, the root mean square error (*RMSE*) and mean coefficient of determination (*R*^2^) were introduced to quantify the prediction accuracy of the proposed method. *RMSE* represents the degree of fitting between the predicted value and the observed value curve. The closer the *RMSE* is to 0, the better the prediction performance will be. The calculation formula is as follows:

(3) $$RMSE=\sqrt{\frac{1}{N}\sum_{i=0}^{N}{\left(y_{out}(i)-y_{real}(i)\right)}^{2}}$$

The closer *R*^2^ is to 1, the more resistant the model is to interference. Usually, an *R*^2^ value greater than 0.8 indicates a good model fit. The formula is as follows:

(4) $$R^{2}=1-\frac{\sum_{i=0}^{N}{\left(y_{out}(i)-y_{real}(i)\right)}^{2}}{\sum_{i=0}^{N}{\left(y_{real}(i)-{\bar{y}}_{real}\right)}^{2}}$$

where *N* represents the total number of samples, *i* represents the index value of the samples, *y_out_* represents the predicted value, and *y_real_* represents the real value.

### 2.4. The TCN-LSTM Hybrid Model

A TCN is essentially a special modification of the CNN architecture to make a convolutional neural network that is more suitable for time series tasks. Currently, TCNs are widely applied and have achieved significant results in various research fields such as wind power forecasting and traffic estimation [32,33]. However, their application in joint angle prediction is relatively rare. In our study, we utilized a TCN to extract deep information from sEMG signals to enhance wrist angle prediction. The main characteristics of the TCN network include three key features: causal convolution, dilated convolution, and residual block [19].

Causal convolution strictly follows the law of causation. The output $c_{t}^{k}$ at time *t* of layer *k* depends only on the data $c_{t}^{k}$~$c_{t-1}^{k-1}$ before time *t* of layer *k* − 1, so no information about the future leaks into the past. For a one-dimensional input sequence $X=(x_{1},x_{2},x_{3},\cdot \cdot \cdot ,x_{n})$ and a convolution filter $F=(f_{1},f_{2},f_{3},\cdot \cdot \cdot ,f_{n})$, the causal convolution at *x_t_* can be defined as

(5) $$(F*X)x_{t}=\sum_{K}^{k=1}f_{k}x_{t}-K+k$$

**Figure 4:**
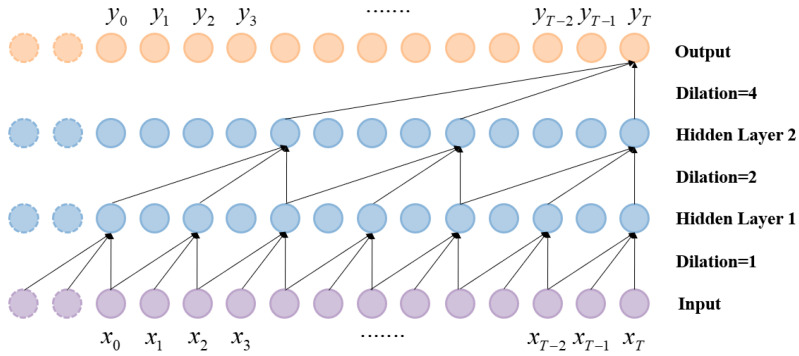
Convolution architecture of a TCN.

However, the length of the history sequence processed by causal convolution is limited, and when processing tasks with longer history records, multiple layers or large convolution cores are required to increase the convolution’s receptive field, increasing the computational burden. Based on this, researchers proposed using dilated convolution to address the problem [34]. Dilated convolution involves skipping some input elements to enable filters to be applied over a larger region than their actual size. The larger receptive field means that more historical data can be captured with fewer parameters and fewer hidden layers [35]. As illustrated in Figure 3, larger filters were generated from the original filter by introducing zeros. This can be mathematically expressed using the following formula:

(6) $$(F*X)x_{t}=\sum_{K}^{k=1}f_{k}x_{t}-(K-k)*d$$

where *d* is the dilation rate, which usually takes a value of 1, 2, 4, 8, etc. (2 to the power) and k is the convolution kernel size. The architecture of a dilated causal convolution is shown in Figure 4.

**Figure 5:**
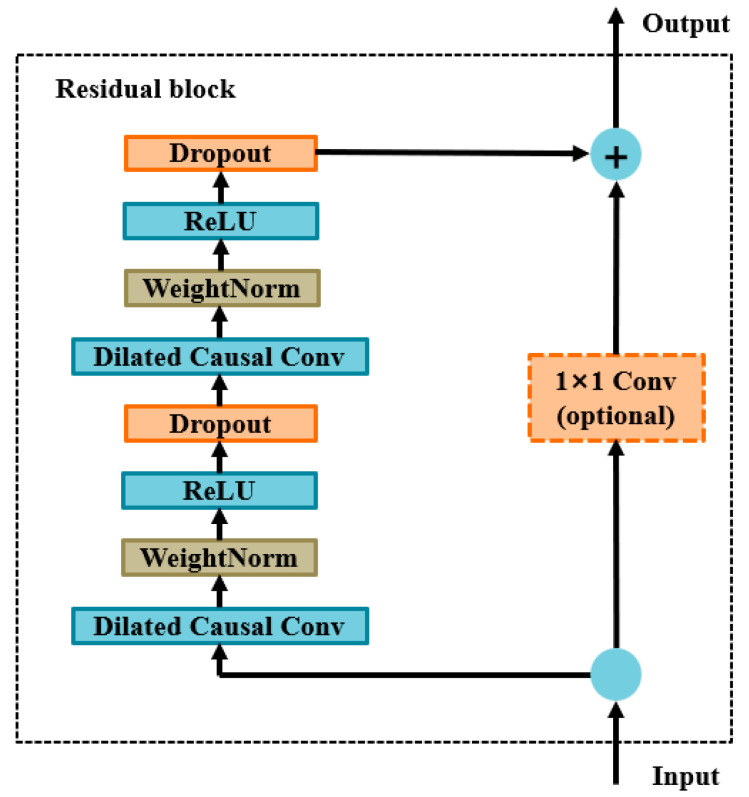
Residual connection block of the TCN.

When dealing with complex prediction tasks such as long sequences, this particular convolutional structure requires continuous updating of weight parameters, leading to an increase in the computational cost of the network. Therefore, the residual connection is used to replace the last convolution layer in the TCN, and the deep features extracted by the TCN are fused with the original manual features to realize the cross-layer transmission of the network, which not only reduces the operating cost of the network but also effectively solves the problems of gradient disappearance and explosion [36]. This structure is shown in Figure 5.

**Figure 6:**
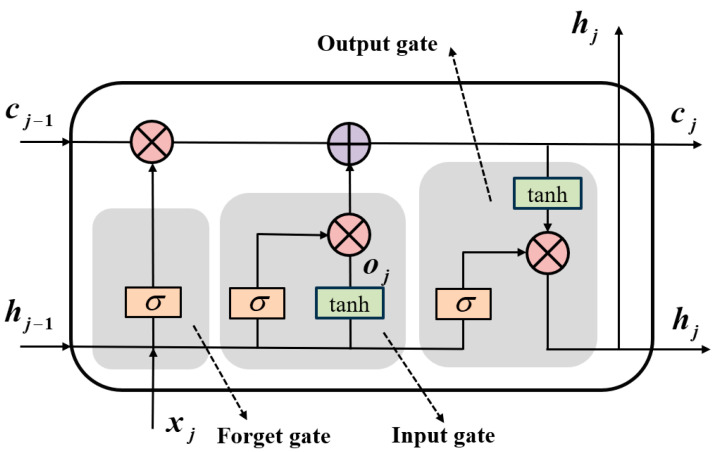
The architecture of the LSTM network unit.

LSTM is a kind of network that can capture long-term dependencies in time series by modifying hidden layer neurons based on a RNN [37,38]. This advantage is contingent upon the cell state and the gated structure. In Figure 6, the fundamental composition of the hidden layer in LSTM is illustrated. $c_{j}$ represents the activation vector, $h_{j}$ signifies the hidden state, and the gating structure encompasses an input gate that regulates the information to be added to the cell state at the current time. Additionally, there is a forget gate that determines which information from the cell state at a prior moment should be discarded, and a hidden output gate that governs the information from the cell state to be transmitted to the next moment [39,40]. The update of the LSTM unit at time step j can be described as

(7) $$\begin{array}{l} i_{j}=\sigma (W_{i}[h_{j-1},x_{j}]+b_{i})\\ f_{j}=\sigma (W_{f}[h_{j-1},x_{j}]+b_{j})\\ g_{j}=\tanh(W_{g}[h_{j-1},x_{j}]+b_{j})\\ o_{j}=\sigma (W_{o}[h_{j-1},x_{j}]+b_{o})\\ c_{j}=f_{j}⊙c_{t-1}+i_{j}⊙g_{j}\\ h_{j}=o_{j}⊕\tanh(c_{j}) \end{array}$$

where σ represents the sigmoid function, tanh represents the hyperbolic tangent function, $\left[h_{j-1},x_{j}\right]$ represents the connection between $h_{j}$ and $x_{j}$, and $i_{j}$, $f_{j}$, $g_{j}$, and $o_{j}$ represent the activation vectors of the input gate, the forgetting gate, the cell alternative value, and the output gate, respectively. ⊙ represents element-by-element multiplication, and $W_{j}$, $W_{f}$, $W_{g}$, and $W_{o}$ are the weight matrices on each gate, respectively, to map the input data and previously hidden states to the activation value of the opposite door. $b_{i}$, $b_{j}$, $b_{g}$, and $b_{o}$ are the corresponding paranoia vectors that adjust the activation threshold of the gate.

The integration of a TCN and LSTM offers a synergistic approach to sEMG signal processing. TCNs excel at capturing intricate deep features from sEMG signals, while LSTM effectively handles temporal dependencies within the input data. By combining these strengths, the proposed TCN-LSTM hybrid model facilitates nuanced feature extraction at both the global and local levels. Moreover, LSTM’s regularization capabilities mitigate overfitting concerns arising from excessive TCN layers, endowing the model with enhanced generalization capacity and bolstering its stability and robustness. The TCN-LSTM model is anticipated to perform exceptionally well in wrist angle prediction tasks owing to a number of crucial aspects, the details of which are discussed below.

Firstly, the long-term dependencies and inherent complex patterns in sEMG data are effectively managed by capturing features at different time scales. Additionally, by integrating TCN and LSTM modules, smooth feature extraction can be performed at both the global and local levels of sEMG data, thus facilitating a comprehensive feature learning capability. Furthermore, the model performs splicing of deep and handcrafted features, which improves the capacity to generate expressive outputs. In summary, this fusion not only strengthens the model’s capacity to interpret sEMG data, but also makes it more capable of handling complex sequence patterns and noise interference.

**Figure 7:**
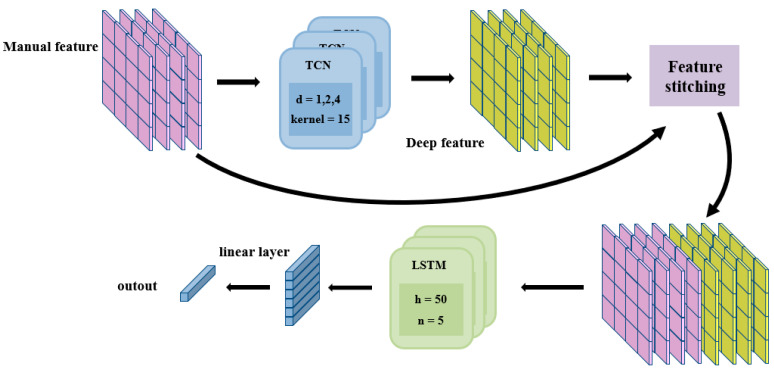
The architecture of the hybrid TCN-LSTM model.

Figure 7 illustrates the comprehensive workflow of the proposed model. Initially, the sEMG signal’s manual features undergo a thorough analysis, employing the TCN in the first step. This involves utilizing a TCN architecture with three layers, with expansion coefficients of 1, 2, and 4, and employing a filter size of 15.

Next, the extracted deep features are fused with the manual features through a spliceover process in the second step. Subsequently, the LSTM regression model is constructed in the third step. Specifically, the LSTM architecture is configured with a 5-layer structure featuring 50 hidden units. Finally, the regression results undergo processing through a linear layer to obtain the ultimate prediction outcomes. The main parameters included a batch size of 32, an epoch of 50, an initial learning rate of 0.01, and a 50% reduction in the learning rate after 10 rounds of training.

## 3. Experimental Results and Discussion

This section presents and analyzes the performance of the proposed method on the public dataset Ninapro and discusses the results compared with that of other models. In addition, we evaluated the effect of different filter sizes on the model’s performance. All the analysis results were based on the movements of the eight subjects detailed in Table 1 and Figure 2. We divided the data for each subject into a training set and a test set in a 1:1 ratio.

### 3.1. Effect of Different Parameters on the Performance of Model Estimation

Using high-frequency original sEMG signals directly as model inputs leads to a huge amount of data for computation, which affects the whole system’s computation time. In addition, there are many redundant parts in the middle of the data. To alleviate the delay between the generation of human movements and the estimation of the system’s angle, a frequently adopted method is used to map the original sEMG signal onto a feature vector that reflects the nature of the signal. By segmenting the original sEMG signal into independent segments, each segment is computed independently to finally obtain the feature vector. In addition, the difference in the size of the time window affects the accuracy of the wrist angle estimation. Choosing too long a time window will lead to the occurrence of delay phenomena, while too short a time window does not reflect the effect of the segmentation time window. Table 3 shows the performance of the model with different window sizes, and the results showed that when the window size was 100 ms, the performance was best. Thus, the 100 ms time window was used in the experiments.

**Figure 8:**
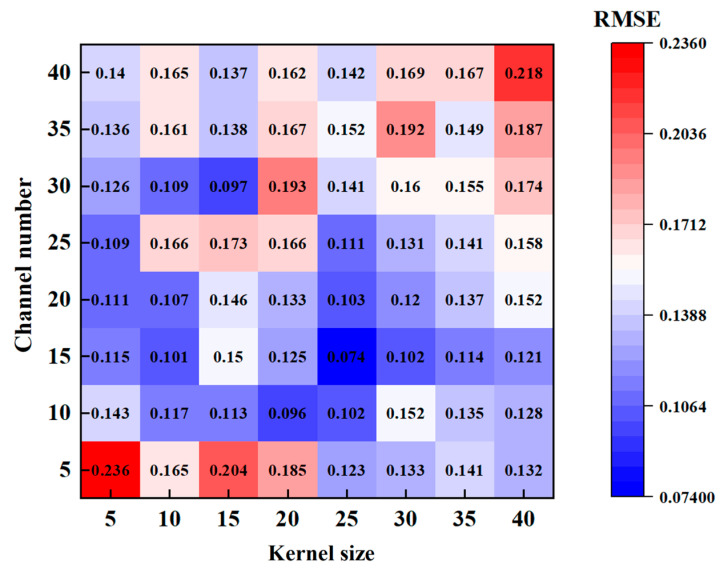
The relationship between *RMSE* and channel number and kernel size.

Selecting the appropriate hyper-parameters plays an important role in deep learning technology. It is not only necessary to select the right learning rate, batch size, and loss function but also to determine the right network hyper-parameters (i.e., kernel size, convolutional channel, discard rate, etc.) [41]. To select the hyper-parameters with the best estimation performance, we altered the convolution kernel size and the number of convolution channels, which have the most significant influence on the model. As shown in Figure 8, we calculated the *RMSE* value of the estimated results from altering the convolution kernel size and the number of convolutional channels from 5 to 40. The different hyper-parameter settings had a significant impact on the estimation performance of the model. Specifically, when the kernel size or the number of convolutional channels remained unchanged, the value of *RMSE* first increased and then decreased with the increase of another parameter. When the kernel size was 15 and the number of convolutional channels was 25, the value of *RMSE* was the smallest. If the kernel size and the number of convolutional channels were too small, the underlying information was lost, and the complete information of the movement could not be captured. If the kernel size was too large, the effective information experienced interference, and the performance of the model declined. Finally, after comparing other relevant factors, we set the convolution kernel size to 15 and the number of convolutional channels to 25 as the optimal hyper-parameters for the current task.

### 3.2. Comparison of Estimation Results of Different Models

**Figure 9:**
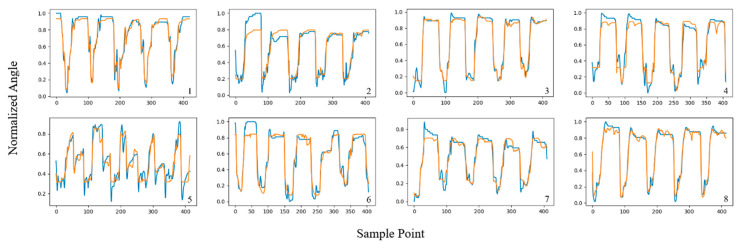
Estimation results of WECH movement in eight subjects using TCN-LSTM model.

**Figure 10:**
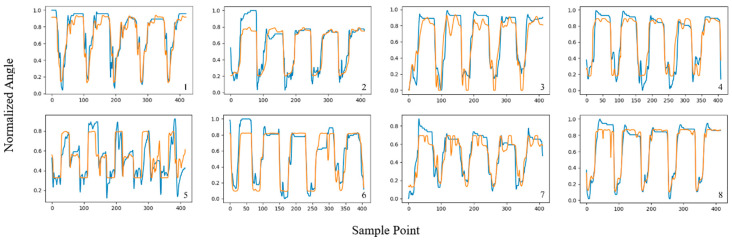
Estimation results of WECH movement in eight subjects using TCN model.

**Figure 11:**
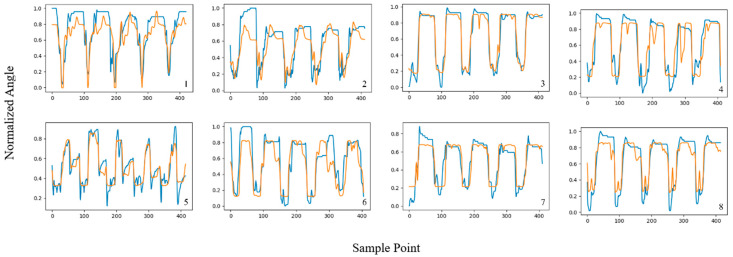
Estimation results of WECH movement in eight subjects using LSTM model.

In this section, the angle estimation performance of the proposed TCN-LSTM hybrid model is studied and compared with that of the other two models. All model training processes were based on the PyTorch 1.7.1 framework; the segmentation of the original sEMG signals and other preprocessing steps were performed using Matlab 2022a; and the processed sEMG signals were input into the TCN, LSTM, and TCN-LSTM networks for training. Figure 9, Figure 10 and Figure 11 shows the estimated results of WECH movement in the eight subjects based on three models; labels 1–8 refer to subjects 1–8, respectively, and each subplot represents the estimation results of the wrist joint, where the *x*-axis represents the sampling point and the *y*-axis is the normalized wrist angle. Each peak and trough represent a movement; the blue line represents the actual angle, and the orange line represents the predicted angle.

It is worth noting that all three models showed good estimation results, which proves the rationality of the previous work (including the selection of features, the partitioning of datasets, the determination of hyper-parameters, etc.). Furthermore, among the three models, the TCN-LSTM hybrid model had the best fitting effect between the estimated value and the real value, and the estimated curve was clearly more consistent with the actual curve than the other two. We also found that the motion estimation effect of subject 5 was not very good under the TCN and LSTM models; one reason is due to individual differences. In general, differences in body structure will affect the acquisition of sEMG, thus increasing the complexity of motion estimation [42]. Another reason is that the angle data collected from subject 5 did not approximate a simple periodic signal, and it is difficult for a single network to obtain a good estimation result. Nevertheless, the proposed TCN-LSTM model performed well in motion estimation for all subjects, demonstrating the robustness and generality of the proposed method.

### 3.3. Comparison and Analysis of the Performance of Different Models

The *RMSE* values for motion estimation across three movements (WF, WUD, and WECH) for the eight subjects using the TCN, LSTM, and TCN-LSTM models are shown in Table 4. Inputting the preprocessed and feature-extracted sEMG signals into these models yielded WF, WUD, and WECH *RMSE* values of 0.1086, 0.1151, and 0.1033 for the TCN model, and 0.1357, 0.1355, and 0.1323 for the LSTM model, respectively. Meanwhile, the TCN-LSTM model achieved RMSE values of 0.0640, 0.0658, and 0.0625 for WF, WUD, and WECH movements, demonstrating a reduction in *RMSE* of approximately 51% compared to the TCN model and about 42% compared to the LSTM model. This highlights that the TCN-LSTM model exhibits significantly enhanced predictive and fitting capabilities for motion estimation compared to the TCN and LSTM models.

Table 5 summarizes the *R*^2^ evaluation results for motion estimation across the three movements (WF, WUD, and WECH) using the TCN, LSTM, and TCN-LSTM models with input from preprocessed and feature-extracted sEMG signals. The TCN model achieved significantly higher *R*^2^ values (WF: 0.815, WUD: 0.8077, WECH: 0.8396) compared to the LSTM model (WF: 0.7636, WUD: 0.7482, WECH: 0.7802), but were lower compared to the TCN-LSTM model (WF: 0.9286, WUD: 0.9225, WECH: 0.9377). Moreover, the *R*^2^ values obtained by the TCN-LSTM model were closer to 1, indicating that the estimation results were more closely aligned with the actual angle.

The research results showed that the *RMSE* and *R*^2^ values for WECH movements were the best among the three movements selected in this study, which was largely related to the hand position recorded by the angle data we selected.

**Figure 12:**
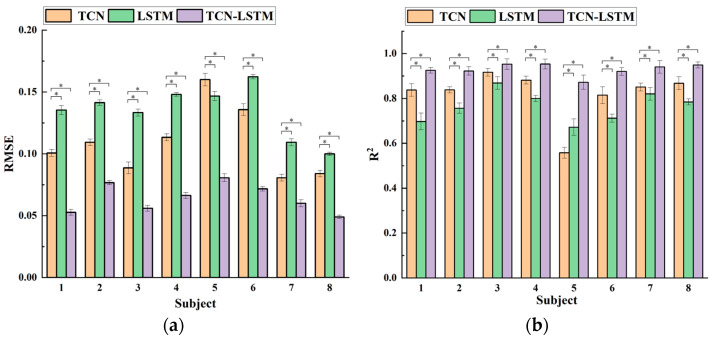
The histogram of the average performance for the selected movement types in the 8 subjects. (**a**) Average *RMSE* results for the three movements using the three models. (**b**) Average *R*^2^ results for the three movements using the three models. ‘*’ indicates statistical significance (*p* < 0.05).

Figure 12 depicts the average *RMSE* and *R*^2^ values across all the movement types for the eight participants. For the participants, the TCN-LSTM model exhibited the lowest *RMSE* and highest *R*^2^ values, convincingly demonstrating its superior performance compared to the other two models. The *RMSE* values for all the models across the eight participants were below 0.17, and the *R*^2^ values were above 0.5, affirming the feasibility of using the RMS and MAV features as inputs. Furthermore, we observed that for participant 5, the LSTM model exhibited a lower *RMSE* and higher *R*^2^ compared to the TCN model. This can be attributed to the dataset of subject 5 showing strong non-periodic characteristics, where the recursive structure of the LSTM model better accommodates such specific conditions compared to the convolutional structure of the TCN model. The effect of the neural network model on the estimated accuracy was investigated by using analysis of variance (ANOVA). A *p* value of less than 0.05 was considered statistically significant. In the ANOVA test, the *RMSE* for the TCN-LSTM model (0.064 ± 0.0022) was less than that of the TCN (0.109 ± 0.0035, *p* < 0.05) and LSTM models (0.135 ± 0.0025, *p* < 0.05). The *R*^2^ with the TCN-LSTM model (0.93 ± 0.021) was higher than that of the TCN (0.821 ± 0.023, *p* < 0.05) and LSTM models (0.764 ± 0.024, *p* < 0.05). This ANOVA test illustrates that the TCN-LSTM hybrid model significantly outperformed the TCN model and the LSTM model in terms of estimation accuracy.

**Figure 13:**
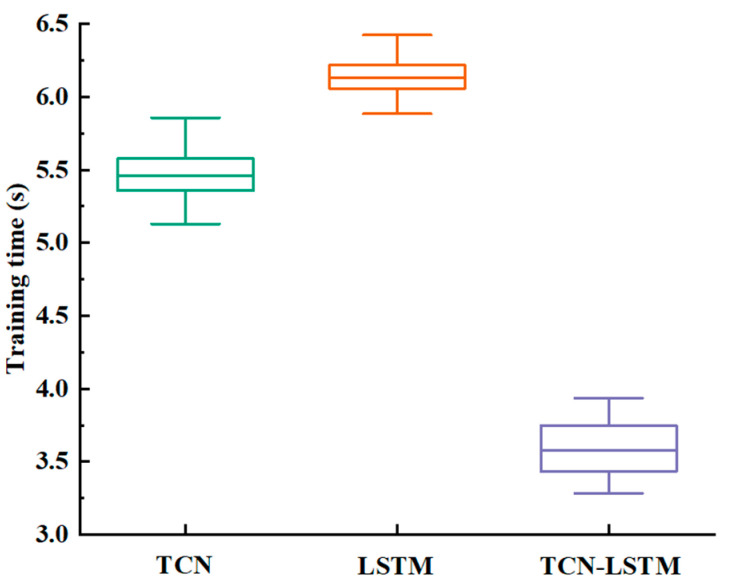
Comparison of training time of the three models.

Figure 13 depicts the training times required for the three models under identical settings (batch size, learning rate, and epochs). The TCN-LSTM model exhibited significantly shorter training times compared to both the TCN and LSTM models. Shorter training times lead to faster system response speeds, which is crucial for meeting the real-time requirements of continuous motion estimation.

**Figure 14:**
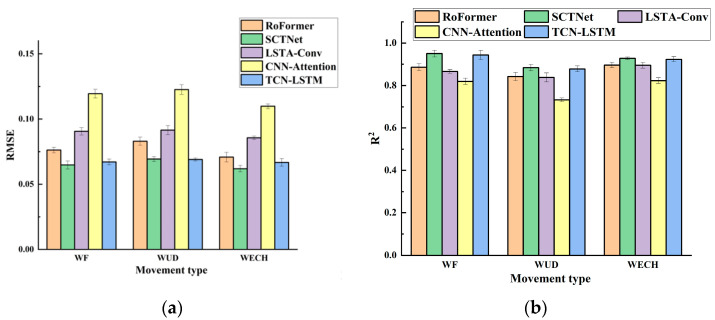
Histogram of the average performance of 5 models for different movement types. (**a**) Average *RMSE* results of five models for WF, WUD, and WECH. (**b**) Average *R*^2^ results of five models for WF, WUD, and WECH.

Furthermore, we compared our proposed method to other advanced models proposed in recent studies based on the Ninapro dataset, such as RoFormer, SCTNet, LSTA-Conv, and CNN-Attention. Table 6 shows the overall performance of the five models on continuous motion estimation of the wrist joint, and Figure 14 shows the average performance of these models for each movement type, demonstrating that the TCN-LSTM model outperformed most models. Although the TCN-LSTM model’s *RMSE* and *R*^2^ performance were not as good as those of SCTNet, its real-time capability was significantly better, and we presume that this is due to the attention mechanism of SCTNet, which improves model performance while increasing the time consumption of the model inference process.

In this section, we first explored the optimal kernel size and convolution channel for the model. Subsequently, the preprocessed and feature-extracted sEMG signals were fed into the TCN, LSTM and TCN-LSTM models. The study recorded the motion estimation graphs, *RMSE* and *R*^2^ values, and time consumed for training for each model. Through several evaluations, it was found that all three models were able to estimate the wrist angle based on the sEMG signal, and the performance of the proposed TCN-LSTM model was the best among the three. The statistical analysis shows that the average *RMSE* of the estimations obtained by the TCN-LSTM model was 0.064, which significantly outperformed the TCN (0.109, *p* < 0.05) and LSTM models (0.135, *p* < 0.05). Moreover, the average *R*^2^ value for the TCN-LSTM model (0.93) was the highest among all the models, indicating its strong anti-disturbance capabilities.

## 4. Conclusions

In this study, we proposed a TCN-LSTM hybrid model based on sEMG signals for accurate and flexible joint angle estimation. We validated the performance and capabilities of our proposed model on the publicly available Ninapro dataset. Comprehensive comparisons with related models demonstrated the robustness of the TCN-LSTM model over the TCN and LSTM models in joint angle estimation. The statistical results indicated that the TCN-LSTM model achieved an average *RMSE* of 0.064, representing a 41% reduction compared to the TCN model and a 52% reduction compared to the LSTM model. The TCN-LSTM model also achieved an average *R*^2^ of 0.93, indicating an 11% improvement over the TCN model and an 18% improvement over the LSTM model. Additionally, the TCN-LSTM hybrid model exhibited the shortest training time among the three models. These results indicate that the TCN-LSTM hybrid model not only accurately estimates joint angles with a relatively stable performance, but that it also excels in real-time applications. This is attributed to its ability to extract deep information from sEMG signals and capture long-term dependency relationships.

Future research directions include testing on a wider variety of and more complex movements to ensure applicability across diverse scenarios. Additionally, optimizing the internal details of the model is essential to enhancing its real-time estimation performance.

## Figures and Tables

**Table 1:** Detailed physiological information about the 8 subjects.

Subject	Gender	Age	Height	Weight
S1	Male	31	170	75
S2	Male	27	170	62
S3	Male	22	180	85
S4	Male	27	183	95
S5	Male	27	178	75
S6	Female	22	163	48
S7	Male	28	170	60
S8	Female	27	164	54

**Table 2:** Performance comparison of the six features.

Feature	MAV	RMS	VAR	MNP	MNF	MDF
*RMSE*	0.0712	0.0703	0.0722	0.0691	0.0709	0.0704
*R* ^2^	0.918	0.918	0.915	0.922	0.920	0.920
Training time (ms)	2792	2412	2816	7545	6970	7663

**Table 3:** The performance of the TCN-LSTM model with different window sizes.

Window Length (ms)	Mean *RMSE*	Mean *R*^2^
50	0.097 ± 0.012	0.859 ± 0.012
100	0.064 ± 0.008	0.918 ± 0.011
150	0.088 ± 0.011	0.905 ± 0.022
200	0.146 ± 0.023	0.768 ± 0.15

**Table 4:** *RMSE* results for wrist angle prediction in 8 subjects using the three models.

Subject	TCN			LSTM			TCN-LSTM		
	WF	WUD	WECH	WF	WUD	WECH	WF	WUD	WECH
1	0.092	0.122	0.088	0.128	0.131	0.147	0.051	0.058	0.049
2	0.11	0.109	0.109	0.144	0.124	0.156	0.061	0.078	0.091
3	0.094	0.098	0.074	0.13	0.137	0.133	0.067	0.052	0.049
4	0.095	0.128	0.117	0.148	0.155	0.141	0.073	0.056	0.07
5	0.148	0.166	0.166	0.151	0.163	0.126	0.079	0.098	0.065
6	0.15	0.139	0.118	0.164	0.157	0.166	0.049	0.091	0.075
7	0.092	0.062	0.088	0.129	0.122	0.077	0.076	0.049	0.055
8	0.088	0.097	0.067	0.092	0.095	0.113	0.056	0.045	0.046
Average	0.10862	0.11512	0.10337	0.13575	0.1355	0.13237	0.064	0.06587	0.0625

**Table 5:** *R*^2^ results for wrist angle prediction in 8 subjects using the three models.

Subject	TCN			LSTM			TCN-LSTM		
	WF	WUD	WECH	WF	WUD	WECH	WF	WUD	WECH
1	0.768	0.861	0.885	0.579	0.828	0.685	0.883	0.93	0.963
2	0.891	0.794	0.831	0.827	0.787	0.656	0.966	0.919	0.882
3	0.879	0.926	0.946	0.89	0.885	0.832	0.939	0.944	0.976
4	0.912	0.873	0.86	0.855	0.748	0.797	0.946	0.964	0.95
5	0.561	0.555	0.557	0.59	0.59	0.836	0.877	0.86	0.88
6	0.752	0.848	0.844	0.695	0.748	0.693	0.913	0.913	0.936
7	0.889	0.821	0.842	0.823	0.759	0.88	0.948	0.935	0.939
8	0.868	0.784	0.952	0.85	0.641	0.863	0.957	0.915	0.976
Average	0.815	0.80775	0.83962	0.76362	0.74825	0.78025	0.92862	0.9225	0.93775

**Table 6:** Overall performance of five models on continuous motion estimation of wrist joint.

Model	RoFormer [25]	SCTNet [43]	LSTA-Conv [44]	CNN-Attention [45]	TCN-LSTM
*RMSE*	0.0767 ± 0.0079	0.0653 ± 0.0037	0.0892 ± 0.0076	0.1173 ± 0.0056	0.0676 ± 0.0036
*R* ^2^	0.875 ± 0.052	0.921 ± 0.028	0.866 ± 0.041	0.791 ± 0.084	0.915 ± 0.018
Times (ms)	8.528	10.68	5.597	27.22	3.463

## Data Availability

The data in this study are a portion of the publicly available dataset called Non-Invasive Adaptive Prosthetics (Ninapro) subset DB1.
